# Ertapenem Resistance of *Escherichia coli*

**DOI:** 10.3201/eid1302.060747

**Published:** 2007-02

**Authors:** Marie-Frédérique Lartigue, Laurent Poirel, Claire Poyart, Hélène Réglier-Poupet, Patrice Nordmann

**Affiliations:** * Université Paris XI, Kremlin-Bicêtre, France; † Université Paris V, Paris, France

**Keywords:** Ertapenem resistance, Escherichia coli, CTX-M, extended-spectrum β-lactamase, outer membrane protein, dispatch

## Abstract

An ertapenem-resistant *Escherichia coli* isolate was recovered from peritoneal fluid in a patient who had been treated with imipenem/cilastatin for 10 days. Ertapenem resistance may be explained by a defect in the outer membrane protein and production of extended-spectrum β-lactamase CTX-M-2.

Of all β-lactam antimicrobial drugs, carbapenems (imipenem, meropenem, and ertapenem) have the most consistent activity against *Enterobacteriaceae*. Activity is retained against most isolates that produce high-level AmpC β-lactamases (cephalosporinases) and clavulanic-acid–inhibited extended-spectrum β-lactamases (ESBL) (*1*). However, a few carbapenem-resistant enterobacterial isolates have been reported; resistance may be caused by production of carbapenemases (*2*) or by combined mechanisms of an outer membrane permeability defect and extended-spectrum β-lactamases or cephalosporinase (*3*–*6*). Spread of CTX-M type ESBLs, especially in *Escherichia coli,* may provide a favorable background for selection of carbapenem resistance. Resistance to the recently introduced ertapenem has not been reported in *E. coli* associated with a CTX-M-type enzyme. We describe the clinical and microbiologic features associated with an ertapenem-resistant *E. coli* isolate that had reduced susceptibility to imipenem after in vivo treatment with imipenem/cilastatin and provide a detailed molecular analysis of the antimicrobial drug resistance mechanisms.

## The Study

*E. coli* CO strain was recovered from a 50-year-old immunocompromised woman who was hospitalized for a combined liver and heart transplant. She had a history of cardiac failure, hepatitis C virus–related liver cirrhosis, and chronic renal insufficiency. After surgery, septic shock developed related to perforation of the colon. The patient received a full dose of imipenem/cilastatin (2 g/day), a reduced dose of vancomycin (400 mg/day), gentamicin (100 mg/day for 2 days), and fluconazole (100 mg/day). Ten days later, peritoneal lavage and surgery to remove diseased colonic tissue were performed, but the patient died 2 days after surgery. Culture of the peritoneal fluid yielded an ertapenem-resistant *E. coli* CO strain.

Disk diffusion susceptibility testing with antimicrobial drug–containing disks (Sanofi Diagnostics Pasteur, Marnes-La-Coquette, France) (*7*) was performed with and without cloxacillin (250 mg/L), which is a β-lactam molecule that inhibits in vitro cephalosporinase activity (*5*). MICs were determined by an agar dilution technique and interpreted according to Clinical and Laboratory Standards Institute guidelines (*7*). The *E. coli* CO strain was resistant to extended-spectrum cephalosporins, cefoxitin, and moxalactam. In addition, it was intermediately susceptible to imipenem and meropenem (MIC 8 mg/L each) and was resistant to ertapenem (MIC >256 mg/L) (Table). The *E. coli* CO strain was also resistant to gentamicin, kanamycin, chloramphenicol, tetracycline, and trimethoprim-sulfamethoxazole; intermediately susceptible to nalidixic acid and tobramycin; and remained susceptible to amikacin, netilmicin, ofloxacin, and ciprofloxacin. Antimicrobial drug susceptibility testing on cloxacillin-containing plates indicated absence of consequential cephalosporinase activity. However, the ceftazidime/clavulanic acid synergy test result was slightly positive. A β-lactamase extract from a culture of *E. coli* CO subjected to isoelectric focusing analysis showed 3 β-lactamase activities with pI values of 5.4, 6.1, and 7.9 (*8*). This extract did not hydrolyze carbapenems according to spectrophotometer measurements (*8*). Conjugation experiments that used an azide-resistant *E. coli* J53 strain as recipient strain (*5*), followed by selection on Mueller-Hinton agar plates containing 100 mg/L sodium azide and 100 mg/L amoxicillin or 2 mg/L of cefotaxime, yielded transconjugants. Two conjugative plasmids (pCO-1, 160 kb; pCO-2, 150 kb) were extracted from those transconjugants by the Kieser technique (*5*). They conferred resistance to amoxicillin and ticarcillin, whereas pCO-1 conferred additional resistance to extended-spectrum cephalosporins (Table). These transconjugants were fully susceptible to carbapenems. Standard PCR conditions were used to amplify several β-lactamase genes coding for carbapenemases (*bla*_KPC_, *bla*_NMC-A_); extended-spectrum β-lactamases including *bla*_TEM_, *bla*_SHV_, *bla*_CTX-M_, *bla*_VEB_, *bla*_PER_; and oxacillinases (OXA-1, OXA-2, OXA-10, OXA-21, and OXA-48) (*2*,*9*). PCR amplification and sequencing identified an extended-spectrum β-lactamase *bla*_CTX-M-2_ gene located on plasmid pCO-1, whereas a *bla*_TEM-1_ gene that coded for narrow-spectrum penicillinase and a *bla*_OXA-10_ gene that coded for oxacillinase were both located on a 150-kb plasmid pCO-2. The surrounding regions of the *bla*_CTX-M-2_ gene corresponded to those of a *sul1*-type class 1 integron. This gene was bracketed by a duplication of the 3′-conserved sequence region of the class 1 integron and was not associated with a 59-bp element. The common region open reading frame (ORF) 513 was found upstream of the *bla*_CTX-M-2_ gene (data not shown) (*10*).

**Table:** MICs of β-lactam antimicrobial drugs for *Escherichia coli* CO, transconjugants pCO-1 and pCO-2, and reference strain *E. coli* J53*

β-lactam	MIC (mg/L)			
	*E. coli* CO	Transconjugant pCO-1 (CTX-M-2)	Transconjugant pCO-2 (OXA-10, TEM-1)	*E. coli* J53
Amoxicillin	>256	>256	>256	2
Amoxicillin + CLA	>256	8	64	2
Ticarcillin	>256	>256	>256	2
Ticarcillin + CLA	>256	32	128	2
Piperacillin	>256	>256	64	1
Piperacillin + TZB	256	2	32	1
Cephalotin	>256	>256	4	4
Cefoxitin	256	4	4	4
Ceftazidime	64	4	0.06	0.06
Ceftazidime + CLA	1	0.125	0.06	0.06
Cefotaxime	>256	64	0.06	0.06
Cefotaxime + CLA	128	1	0.06	0.06
Cefepime	>256	16	0.06	0.06
Moxalactam	128	0.06	0.06	0.06
Aztreonam	>256	32	0.06	0.06
Imipenem	8	0.25	0.25	0.25
Meropenem	8	0.06	0.06	0.06
Ertapenem	>256	0.03	0.03	0.03

*CLA, clavulanic acid at a fixed concentration of 2 mg/L; TZB, tazobactam at a fixed concentration of 4 mg/L..

The outer membrane protein (OMP) profiles of *E. coli* isolates were extracted and analyzed by using sodium dodecyl sulfate–polyacrylamide gel electrophoresis, as described (*4*,*5*,*11*,*12*) and compared with profiles of *E. coli* control strains expressing porins OmpC or OmpF (*13*). The OMP profiles of *E. coli* CO showed expression of OmpA and OmpF and no expression of OmpC (Figure). Using whole-cell DNA of *E. coli* CO as a template and primers EcOmpFA (5′-CAGGTACTGCAAACGCTGC-3′) and EcOmpFB (5′-GTCAACATAGGTGGAC ATG-3′) that anneal at the ends of the *ompF* gene of *E. coli* (*5*), we obtained a 953-bp internal fragment of the *ompF* gene (data not shown). Sequencing identified a wild-type *ompF* gene. When primers EcOmpCA (5′-GTTAAAGTACTGTCCCTCCTG-3′) and EcOmpCB (5′-GAACTGGTAAACCAGACCCAG-3′) were used, no amplification was obtained for *E. coli* CO, whereas a 1,086-bp internal fragment of the *ompC* gene of the *E. coli* control strain expressing OmpC (*10*) and of 3 wild-type *E. coli* strains was amplified (data not shown). Thus, the *ompC* gene was either disrupted or not present, which explains lack of expression of this protein and might contribute substantially to ertapenem resistance of *E. coli* CO.

**Figure:**
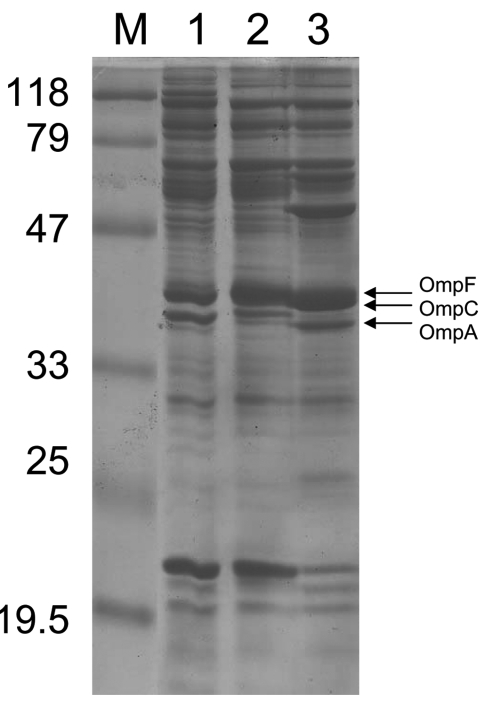
Outer membrane protein (OMP) profiles of *Escherichia coli* strains. OMP content was determined by using sodium dodecyl sulfate–polyacrylamide gel electrophoresis. Lane 1 corresponds to *E. coli* CO clinical isolate; lane 2, *E. coli* JF 568 strain expressing OmpC; lane 3, *E. coli* JF 701 strain lacking OmpC (*9*). The molecular mass marker (M) and corresponding sizes (in kilodaltons) are indicated on the left. Horizontal arrows on the right indicate positions of the OMPs OmpF, OmpC, and OmpA.

## Conclusions

Ertapenem resistance has been reported in *Klebsiella pneumoniae*–producing CTX-M–type ESBLs that have a permeability defect (*3*,*4*,*14*). We report here the first ertapenem-resistant *E. coli* clinical isolate that produced a CTX-M-type ESBL and that was deficient in porin OmpC. This finding may be clinically relevant because ertapenem is approved for treatment of peritonitis, abdominal infection, and complicated skin and soft tissue infections in patients with diabetes and because *E. coli* is the main species isolated in human infections and the main enterobacterial species that expresses these emerging extended-spectrum β-lactamases CTX-M (*15*). That an imipenem/cilastatin–containing regimen was likely able to select for ertapenem resistance is cause for concern. Moreover, even if the strain is not resistant to imipenem and meropenem, it is no longer totally susceptible. Susceptibility patterns of this *E. coli* CO strain, although resistant to ertapenem, are also highly resistant to extended-spectrum cephalosporins, thereby demonstrating an ESBL phenotype. This information may help with future identification of those multidrug CTX-M (+) resistance isolates for which the best treatment remains carbapenems.
